# Binding modes of diketo-acid inhibitors of HIV-1 integrase: A comparative molecular dynamics simulation study

**DOI:** 10.1016/j.jmgm.2011.04.002

**Published:** 2011-06

**Authors:** Meilan Huang, Guy H. Grant, W. Graham Richards

**Affiliations:** a School of Chemistry and Chemical Engineering, David Keir Building, Queens University Belfast, Stranmillis Road, Belfast BT9 5AG, UK; b Unilever Centre for Molecular Informatics, The University Chemical Laboratory, Lensfield Road, Cambridge CB2 1EW, UK; c Department of Chemistry, Central Chemistry Laboratory, University of Oxford, South Parks Road, Oxford OX1 3QH, UK

**Keywords:** HIV-1 integrase, Diketo acid inhibitors, Molecular dynamics, Ion–pair interaction, Catalytic loop

## Abstract

HIV-1 integrase (IN) has become an attractive target since drug resistance against HIV-1 reverse transcriptase (RT) and protease (PR) has appeared. Diketo acid (DKA) inhibitors are potent and selective inhibitors of HIV-1 IN: however the action mechanism is not well understood. Here, to study the inhibition mechanism of DKAs we performed 10 ns comparative molecular dynamics simulations on HIV-1 IN bound with three most representative DKA inhibitors: Shionogi inhibitor, S-1360 and two Merck inhibitors L-731,988 and L-708,906. Our simulations show that the acidic part of S-1360 formed salt bridge and cation–π interactions with Lys159. In addition, the catalytic Glu152 in S-1360 was pushed away from the active site to form an ion–pair interaction with Arg199. The Merck inhibitors can maintain either one or both of these ion–pair interaction features. The difference in potencies of the DKA inhibitors is thus attributed to the different binding modes at the catalytic site. Such structural information at atomic level, not only demonstrates the action modes of DKA inhibitors but also provides a novel starting point for structural-based design of HIV-1 IN inhibitors.

## Graphical abstract

**Graphical abstract:**

**Figure:**
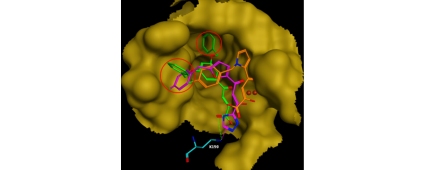

**Highlights:**

► Comparative molecular dynamics simulations on HIV-1 IN bound with L-731,988, L-708,906 and S-1360. ► The acidic end of all the DKA inhibitors studied formed favourable ionic interactions with Lys159. ► The keto–enol parts of these compounds were consistently coordinated to Mg. ► The catalytic residue Glu152 formed a favourable ion–pair interaction with the negatively charged Arg199 on α6 in the most potent DKA inhibitors. ► The complexation with Merck inhibitors and S-1360 significantly constrained the flexible surface loop into an extended or open conformation.

## 1 Introduction

The human immunodeficiency virus type 1 (HIV-1) encodes three enzymes: reverse transcriptase (RT), protease (PR), and integrase (IN). With the emerging drug-resistance of the first two targets, it is urgent to develop potent inhibitors for the third target, IN [1–5]. IN is involved in the viral replication process which undergoes two separate reactions. In the first step, IN processes the viral DNA by removing a conserved dinucleotide from each 3′ end, termed 3′-processing. Then the processed viral DNA is linked to the host cell DNA; the so-called strand transfer reactions [6].

**Fig. 1:**
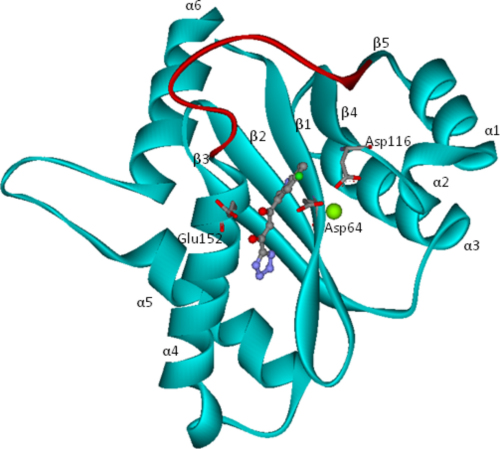
Ribbon diagram of HIV-1 IN (bound with 5-ClTEP). The catalytic DDE triad and ligand 5-ClTEP are shown in ball-and-stick representation. The flexible loop region (residues 138-149) is shown in red.

HIV-1 IN is composed of three functional domains. The N-terminal domain is comprised of residues 1–50 and the catalytic domain comprises amino acids 50–212. The C-terminal domain, which comprises residues 212–288 was suggested to serve in binding the viral DNA during the integration process [7]. Each domain can form dimers and full-length IN is known to act as a multimer [8,9]. Although the respective domain structure has been disclosed, the full length protein has not been resolved. The catalytic domain contains three conserved amino acids, Asp64, Asp116 and Glu152 (Fig. 1), comprising a DDE motif, which is also present in other DNA-processing enzymes such as ASV [10], RHase and Tn5 [11]. In the IN catalytic domain an Mg ion is coordinated to the oxygen atoms of Asp64 and Asp116. It has been suggested that a second metal ion, coordinated to Glu152 and Asp64, is present when binding with the DNA substrate [12,13], although the exact location of the second Mg is not known. A flexible loop, comprising residues 138–149, is known to be crucial for the catalytic activity of HIV integrase [14] and amino acids Lys156, Lys159, Tyr143 and Gln148 are implicated in the binding of viral DNA [15,16].

**Fig. 2:**
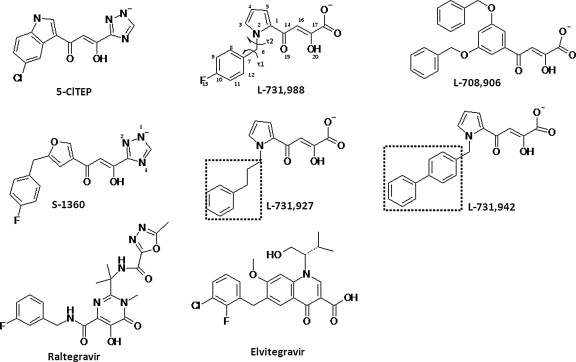
The structures of 5-ClTEP, L-731,988, L-708,906, L-731,927, L-731,942, S-1360, Raltegravir and Elvitegravir. *τ*1 and *τ*2 represent the rotations around the methylene group of L-731,988 are labeled by arrows.

Among the emerging HIV-1 IN inhibitors, 4-aryl-2,4-dioxobutanoic acid derivatives were found to have potent inhibitory activity on strand transfer. Two representative compounds are L-731,988 and L-708,906 [17] (Fig. 2) from Merck. S-1360 [18], a potent IN inhibitor from Shionogi, also belongs to the diketo acid inhibitor family because the trizole part of S-1360 can be regarded as bioisostere of carboxlate (Fig. 2). It failed at phase II clinical trial due to rapid metabolism. Individual or combined mutation of T66I, M154I and S153Y confers resistance for the Merck inhibitors [17] and the mutation of N155S displays cross-resistance for the Merck inhibitors and L-870,810 [19] (a potent strand transfer inhibitor of IN which was derived from pharmacophore search based on the DKA inhibitors but also failed at clinical trial). Nine combined mutations which include T66I, Q146K, S153A and others, all confer resistance to S-1360, indicating that these compounds bind at, or near, the enzyme active site [20]. Two DKA derivatives Raltegravir (MK-0518) [21] and Elvitegravir (GS-9137) have been recently approved as anti-AIDS drug or in clinical trials [22]. Q148K and T66I conferred the highest resistance to both drugs while S153Y conferred relatively greater resistance to Elvitegravir than Raltegravir [23]. Mutation of Tyr143 is known to confer resistance to Raltegravir [24]. The drug resistance to Raltegravir conferred by mutation of N155H, was attributed to the disruption of the metal cofactors with the catalytic carboxylate at the active centre of IN [25]. The mutations of IN which confer resistance to DKA inhibitors and the derivative drugs are listed in Table S1.

The first HIV-1 IN inhibitor complex solved (PDB code: 1QS4 [26]) contains a Shionogi inhibitor 5-ClTEP, 1-(5-chloroindol-3-yl)-3-hydroxy-3-(2H-tetrazol-5-yl)-propenone (Fig. 1). The inhibitor 5-ClTEP in HIV-1 IN is found to stabilize the flexible loop. In the March issue of Nature 2010, the crystal structure of full length prototype foamy virus (PFV) IN in complex with DNA and strand transfer inhibitors Raltegravir and Elvitegravir [27] was reported by Peter Cherepanov. Recently resolved NMR solution conformation of HIV-1 IN disclosed strikingly similarity to the crystal structure of the PFV bound to DNA [28]. However, the HIV-1 IN-DNA structures in presence of the DKA inhibitors such as the Merck Inhibitors and S-1360 have not been resolved.

Computational modelling has been applied to investigate the structures of HIV-1 IN-inhibitor complexes. Docking studies on IN showed that the position of the 5-ClTEP may be affected by crystal packing effects [29]. Flexible docking was performed by Keseru and Kolossvary on 5-ClTEP and L-731,988 [30]. In the past several years, molecular dynamics simulations have been applied to the native and mutated IN [31–33] and IN in complex with 5-ClTEP and a Merck inhibitor L-731,988 [34–37]. Hybrid QM/MM methods were also used to investigate the dynamic behaviour of 5-ClTEP and DKA bound IN [38–41].

Until now, a detailed interaction mechanism between the potent DKA inhibitors and the IN has not yet been completely elucidated, due to unavailability of crystal structures of the IN complexes. In addition, the conformational change which may be associated with DKA binding makes it more complicated to understand the interaction modes. Molecular dynamics simulation is a powerful tool to study the conformational change induced by bound ligands. In the present work, we used *ab initio* calculations to prepare the parameters for the most representative DKA inhibitors; Merck inhibitors L-731,988 and L-708,906, and S-1360.We then performed comparative molecular dynamics simulations of IN in complex with these compounds. The aim of the present work was to explore the binding modes of these representative DKA inhibitors and the induced fit effect of binding on the IN protein structure. Of particular focus is the flexible loop, which is close to the active site. This will uncover structural information for use in structural-based drug design. To our knowledge, our work is the first to compare the binding modes of the representative DKA inhibitors for HIV-1 IN by molecular dynamics simulations.

## 2 Computational methods

### 2.1 Minimum conformations of the L-731,988 anion

The tautomeric forms for the anion of L-731,988 were calculated using the B3LYP/6-31G* method with Gaussian 98 software [42]. Two dihedral rotations around the methylene group of the aromatic part were studied by the potential surface calculated at the HF/STO-3G level followed by single point energy calculations with B3LYP/6-31G* method. The minima obtained were subjected to further optimization at the same level, B3LYP/6-31G** and B3LYP/6-31+G**, followed by a single point energy calculation with B3LYP/6-311+G (3df, 2p).

### 2.2 Building the IN complex structures

The residues Ile141-Asn144 are not determined in the IN-5ClTEP crystal structure (pdb code: 1QS4 [26]) and are only available in two other core domain structures [43,44]. To avoid potential artifact caused by induced fit of ligand binding in the crystal structure of IN bound with 5-ClTEP, the complete core domain of apo IN (pdb code: 1BIS (monomer B)) was used to build the IN in complex with the DKA compounds. Only one metal Mg ion was included in MD simulation, since the position of the second metal ion has not been resolved and it was suggested it may only exist when IN is bound with DNA substrate [12,13]. The Mg ion is built based on the position of Mg in the crystal structure of IN bound with 5-ClTEP. The minimum-energy conformers of the L-731,988 anion were initially docked into the active site of IN, based on the DKA binding model hypothesized by Grobler et al. [12] and also the molecular model where DKA is bound in IN-DNA complex [13] (i.e., the keto–enol oxygen atoms of the DKA inhibitors are coordinated to the Mg ion in our initial models).

We prepared two complex models in which the diketo part of L-731,988 was orientated in two different ways. In the first model, the complex structures were built according to the position and orientation of 5-ClTEP in the crystal structure, with the enol and keto oxygen atoms of the inhibitor coordinated to the Mg ion. We also built a model where two crystal waters (water 44 and 443) coordinated to Mg in the crystal structure of IN-5-ClTEP complex were replaced by the enol and carboxylic oxygen atoms of L-731,988. The carbonyl oxygen of L-731,988 was positioned to interact with the putative second Mg ion between Asp64 and Glu152. The models of IN in complex with another Merck inhibitor L-708,906 and Shionogi inhibitor S-1360 were built by superimposing the keto–enol part of these compounds with that of L-731,988.

### 2.3 Molecular dynamics simulation

Molecular dynamics was run with Charmm [45] (version 33b4, Harvard, Cambridge, MA) using the charmm27 force field [46]. The force field parameters for the ligands were obtained by HF/6-31G* calculation. All crystal water were deleted, polar hydrogen atoms were added according to p*K*_a_ prediction using the electrostatics potential calculation tool Delphi [47], in Biopolymer module of InsightII package (Accelrys, San Diego, CA). Based on the p*K*_a_ calculation, no unusual ionization states were found for the titratable groups (Asp, Glu, Arg, Lys, His, Tyr, Cys and N-ter and C-ter). The hydrogen atoms were minimized with 500 steps of Steepest Descent (SD) optimization; TIP3P water [48] was added in a sphere with a radius of 25 Å around the reaction centre (the Mg ion). Before the unconstrained MD simulation, the solvent was subjected to 1000 steps of SD minimization, and equilibrated for 2.5 ps at 1000 K with solute fixed. The system was then cooled to 300 K and the solvent was equilibrated for additional 15 ps.

MD simulations were performed with a stochastic boundary (SB) setup [49]. Atoms within the sphere of 21 Å (the reaction region) were subjected to a conventional Newtonian dynamics, and atoms in the buffer region (a shell between spheres of radii 21 Å and 25 Å) were treated by Langevin dynamics. Atoms outside were kept fixed. The friction coefficients of 62 ps were applied to the water oxygens and the system was equilibrated at 300 K for 17.5 ps using a time step of 1 fs; the SHAKE algorithm was used to constrain all bonds involving hydrogen atoms. The production simulation was carried out for 10,000 ps (10 ns). Snapshots of the trajectory were taken every 0.25 ps. To obtain the most representative frame from the MD simulations, the average conformation was calculated. This was achieved by averaging the snapshots of the last 500 ps, then choosing a typical structure with the lowest RMSD to the average conformation, and using this in binding mode analysis. This structure was further minimized by Charmm force field.

## 3 Results and discussion

### 3.1 The optimal conformations of 5-ClTEP and L-731,988

The tautomeric conformations of the anionic forms of 5-ClTEP and L-731,988 were considered, starting from their stable neutral enol forms [50]. Given the acidic nature of these compounds (p*K*_a_ is 5 for the tetrazole of 5-ClTEP and 4 for the carboxylic group of L-731,988 [17]), their anionic forms were chosen for the simulation. From our previous calculations for 5-ClTEP and L-731,988 [50], the tautomer with the enol group attached to the carboxylate/tetrazole group has a lower energy than the one with the enol group attached to the pyrrol ring. Because L-708,906 and S-1360 have either a carboxylate or heterozolate acidic group, analogous to L731,988 and 5-ClTEP, respectively, we deemed that they also exist in the tautomeric form with enol connected to the acidic group.

**Fig. 3:**
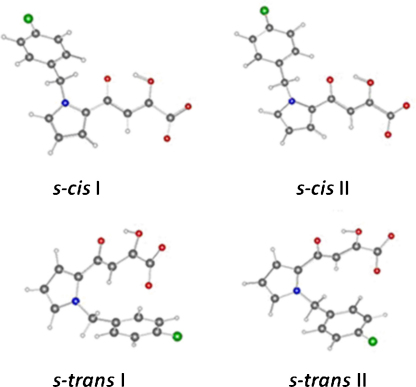
The minimum energy conformations of L-731,988 anions calculated by B3LYP/6-31G* method. a and b are isomers with identical absolute values of *τ* 1 and *τ* 2.

There are two rotatable bonds around the methylene group of the L-731,988 anion, *τ*1 (C_12_C_7_C_6_N_2_) and *τ*2 (C_7_C_6_N_2_N_1_) (Fig. 2). Scanning of these two rotatable bonds with the HF/STO-3G method followed by single point energy calculation with B3LYP/6-31G*, gave two minima including a pair of *s-cis* extended isomers (The pyrrol nitrogen is in the *cis* position of the keto of the diketo acid part, with *τ* 1 of ∓104.5° and *τ* 2 ±72.2°) and a pair of *s*-*trans* folded isomers (*τ* 1 of ∓62.1̊ and *τ* 2 ±105.5°) (Fig. 3). The energy of the *s-cis* isomer is lower than the *s-trans* by 1.43 kcal mol^−1^. Optimization with bigger basis sets B3LYP/6-31G** and B3LYP/6-31+G** gave similar energy differences (1.43 and 1.96 kcal mol^−1^, respectively). Single point energy calculations at high level B3LYP/6-311+G (3df, 2p) based on the B3LYP/6-31+G** optimized geometries were carried out. The relative energy difference remains the same (1.55 kcal mol^−1^). The lower energy of *s-cis* isomers by DFT calculation with different basis set aforementioned indicated that they are more stable than the *s-trans* isomers. Both conformations were used to build the starting internal geometries of the IN-L-731,988 complex.

### 3.2 Mg ion chelation

In the crystal structure of core domain IN in complex with 5-ClTEP, the Mg ion was coordinated to two of the catalytic triad residues, Asp64, and Asp116, and four water molecules. Over the entire simulation of IN in complex with Merck inhibitors or S-1360 (built based on the position and orientation of 5-ClTEP in the crystal structure of IN complex), the carboxylic group of the inhibitors maintained coordination with the Mg ion. Two structural waters were identified, unveiling an octahedral coordination with the Mg ion together with the keto–enol part of the DKA inhibitors and two catalytic triad residues; Asp64 and Asp116 (Supporting information, Fig. S1).

That the keto–enol part of DKA inhibitors was consistently coordinated to the Mg ion supports the hypothesis that DKA compounds inhibit IN by chelating with the metal cofactor [12]. HIV-1 IN is highly conserved with PFV IN at the active site [27]. In the crystal structures of PFV IN bound with two HIV-1 IN drugs, Raltegravir and Elvitegravir, the keto–enol part of these drugs are coordinated to the Mg ion (Fig. S2). Thus, metal chelating modes of DKA compounds is also commensurate with the binding modes of HIV-1 IN drugs with PFV IN, Therefore, the analysis for the MD simulation hereafter will be based on this model.

### 3.3 Dynamic behaviour of the DKA bound IN complexes

**Fig. 4:**
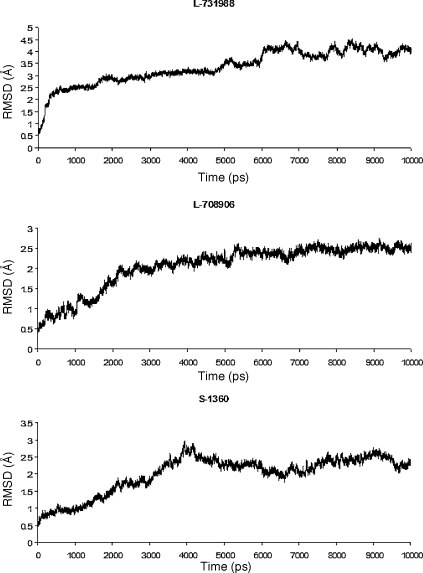
Root-mean-square deviation (RMSD) from the starting structure of the MD simulations, measured for all Cα atoms of the protein IN/DKA.

The overall structural fluctuation was evaluated by analyzing the RMSDs of Cα atom versus simulation time (Fig. 4). All DKA-bound IN complexes reach equilibrium after 5000 ps and maintained at equilibrium for 5000 ps, indicating the systems evolved into stable states and have reasonably converged.

### 3.4 Opening of an additional hydrophobic cavity by L-708,906

**Fig. 5:**
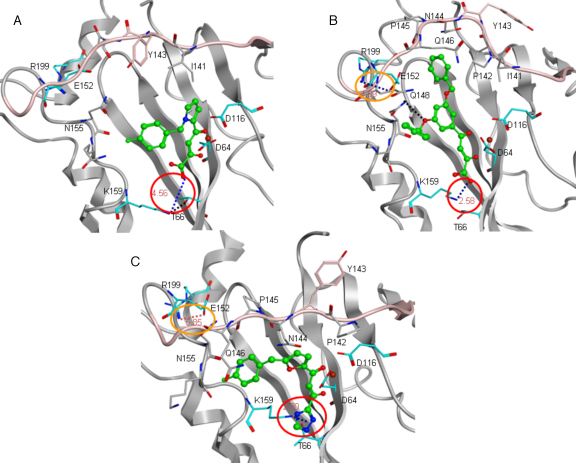
The interaction modes of DKA inhibitors in the active site of HIV-1 IN. The key residues are shown in tube mode and the ligand and Mg ion are shown in stick and ball mode. The surface loop (residues 138-149) is shown in pink by ribbon representation. The salt bridge interactions between ligands and Lys159 are marked in red circles and the ion-pair interactions between Glu152 and Arg199 are circled in orange. (A) L-731,988 (B) L-708,906 (C) S-1360.

We examined the surface around the IN active site where IN is bound with DKA inhibitors. The central pyridine ring of L-731,988 is involved in hydrophobic interactions with Ile141. The aromatic parts of L-731,988 form hydrophobic interactions with Asn155 and Lys156 (Table 1). Over time, the Tyr143 is turned towards the L-731,988, forming additional hydrophobic interaction (Fig. 5). The unique orientation of Tyr143, induced by L-731,988 binding, is favourable to form hydrophobic interaction such that it is likely to be responsible for the potency of L-731,988.

**Fig. 6:**
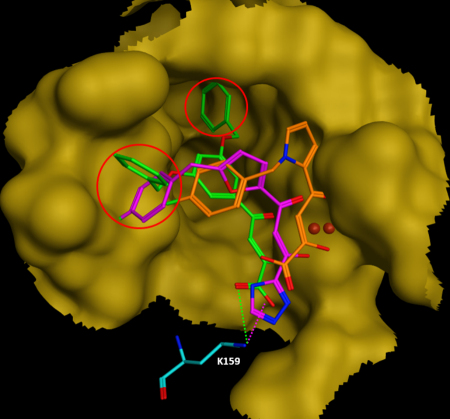
The alignment of the minimized average structures (over last 500ps) of IN-L-708,906 (green), and IN-S-1360 (violet) with IN-L-731,988 (orange). The molecular surface of IN in complex with L-708,906 is shown in gold colour.

Examining of the structure of IN in complex with L-708,906 disclosed that one aromatic ring of L-708,906 is located in the hydrophobic face of a polar cavity formed by Asn155, Lys156 and Gln148. Over time, the catalytic residue Glu152 is pushed away from the Mg ion and the cavity between Ile141 and Gln146 opened, forming an adjacent hydrophobic pocket containing Pro142, Asn144 and Gln146. The second aromatic ring of L708,906 is nested nicely within the second hydrophobic cavity (Fig. 6). The additional hydrophobic interaction between the second benzene ring of L-708,906 with the second hydrophobic cavity thus contributes to its higher potency in IN strand transfer, compared with L-731,988. In addition, the nitrogen atom on the side chain carboxamide of Asn155 forms a H-bond with the oxygen atom of the benzyl ether group of L-708,906. Interestingly, the close contact of DKA inhibitors with Pro145 and Gln146 and the hydrophobic interactions with Pro145 and Tyr143, are also observed in the recently resolved crystal structures of PFV IN bound with diketo-containing HIV-1 IN drugs, Raltegravir or Elvitegravir [27]. These DKA derived IN drugs are in van der Waals contact with Pro214 and Gln215 (corresponding to Pro145 and Gln146 in HIV-1 IN), and form hydrophobic interactions with Pro214 (corresponding to Pro145 and Tyr143 in HIV-1 IN), while the 1,3,4-oxadiazole of Raltegravir forms π–π stacking interaction with Tyr212 (Tyr143 in HIV-1 IN).

Examining of the average structures of S-1360 revealed that the phenyl group of the ligand pointed towards the hydrophobic face of a polar cavity formed by Pro145, Gln146, Asn155 and Lys156 (Fig. S3). The hydrophobic interaction between Gln146 on the flexible loop and the fluorobenzene ring of S-1360 is in agreement with previous experiment where Q146K mutation in combination with other mutations confers resistance to S-1360 [22]. The central furan ring of S-1360 is involved in hydrophobic contact with Pro142.

We then examined the conformational change of IN, in particular the shape of the catalytically active loop region (residues 138–149), in the IN complex structures. It was disclosed that the loop in IN bound with L-731,988 and S-1360, had been constrained into an extended configuration, while the loop in IN bound with L-708,906 is in a more open conformation. This conformation is favourable for the accommodation of an additional aromatic group, such that higher inhibitory activities have been observed for those DKA compounds which bear an additional benzene ring at this position (Fig. 6).

In the crystal structures of apo IN (pdb code: 1BIS or 3L3U), the distance between Thr66 and Lys159 is around 3.0 Å, indicating a H-bond is formed. When IN is bound to 5-ClTEP (pdb code: 1QS4), the distance is increased to 3.7 Å; much too far to form a H-bond. In both IN-L-731,988 and IN-S-1360, Thr66 is brought closer to Lys159, indicating that Thr66 may facilitate the DKA binding through hydrogen bonding with Lys159, which is involved in strong salt ion interaction with the acidic part of the DKA compounds (for further detail refer to the salt bridge section). This is in agreement with mutagenesis experiments where T66I confers inhibitory resistance to this compound [18,22]. However, in IN bound with L-708,906, this H-bond is not observed.

The involvement of interaction between DKA compounds and residues Thr66, Gln146, Gln148, Asn155, Lys156, Lys159 of IN is commensurate with the previous study, where these residues have been suggested to play important roles through affecting IN catalytic activity (Table S1) or cellular DNA binding [15,16].

### 3.5 Salt bridge interactions between the acidic part of DKAs and Lys159

**Fig. 7:**
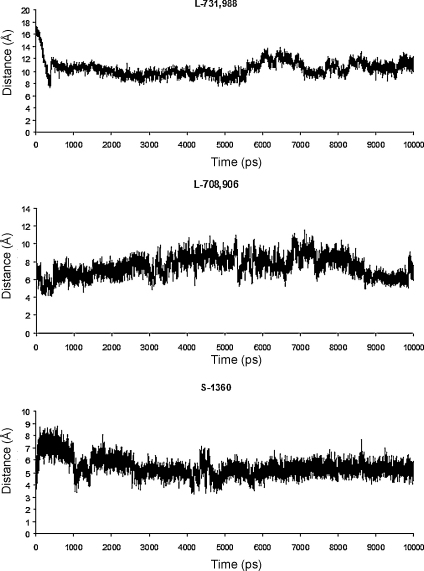
The distance between the ammonia NZ of Lys159 and the acidic oxygen or nitrogen atom of ligand, versus simulation time for the IN in complex with DKA inhibitors.

The time evolution of the distance between the N_Z_ atom of Lys159 and the carboxylate oxygen of Merck inhibitors, or the triazole nitrogen atom of S-1360 is shown in Fig. 7. In the IN bound with 5-ClTEP structure, the distance between Lys159 and the acidic nitrogen of the tetrazole ring of 5-ClTEP is 2.8 Å, which indicates that the ligand forms a salt bridge with Lys159. The distances between Lys159 and the acid part of the Merck inhibitors and S-1360 were examined for the conformation averaged over the last 500 ps of the MD simulations. The residue Lys159 was brought in close proximity to the carboxylate group of L-731,988 with the distance between the ammonia nitrogen of Lys159 and the carboxylate oxygen around 4.5 Å in the IN-L-731,988 complex, implying only a weak salt bridge interaction is formed. In IN bound with L-708,906, the Lys159 was brought even closer to the carboxylate of the ligand and the distance between the ammonia group of Lys159 and the carboxylate group of the ligand was shortened to 2.6 Å, indicating a stronger salt bridge was formed. In the average structures of IN-S-1360 complex the charged ammonia group of Lys159 moved closer to the acidic group of S-1360, with the distance between ammonia nitrogen and triazole nitrogen around 2.9 Å, indicating that a strong salt bridge interaction had formed.

Therefore, a salt bridge interaction is displayed between Lys159 and all the DKA compounds studied, regardless of whether the acidic part is carboxylate group or heterozolate group. Also, a stronger salt interaction was observed for the most potent inhibitors, L-708,906 and S-1360. Thus we suggest that formation of such salt-bridge interaction should to be responsible for the exhibited inhibition activities of these compounds.

In order to assess the angular preference for the perpendicular approach of the positively charged Lys159 to the triazole ring, the distance between the Nz atom of Lys159 and the centroid of the triazole ring was calculated together with the angle formed by Nz of Lys159, the centroid of the triazole ring and N1 atom of S-1360 (Fig. S4). In the minimized average structure over the last 500 ps of simulation, the distance between the Nz of Lys159 to the centroid of the 1, 2, 4-triazole ring is 2.9 Å (Fig. 5) and the angle formed by Nz, the centroid and N1 atom of the triazole is 75°, indicating cation–π interaction also exists for S-1360. Under the physiological condition, S-1360 appears in neutral form as its p*K*_a_ is around 9 [51], therefore for the binding of neutral form of S-1360, the cation–π interaction should be the dominant interaction.

### 3.6 Reorientation of Glu152 to form ionic interaction with R199 and its implication on DKA competitive inhibition

We also examined the effect of inhibitor binding on the orientation of the third catalytic residue, Glu152. When IN is bound with Merck inhibitors and S-1360, Glu152 was pushed away from the catalytic site. When we prepared our manuscript, a high resolution HIV IN crystal structure was resolved, in which the flexible loop displays unique orientation (pdb code: 3L3U) [52]. Of particular interest is the fact that Glu152 is pointing away from the active site, an observation which is in agreement with the reported reorientation of Glu152 in our simulations.

**Fig. 8:**
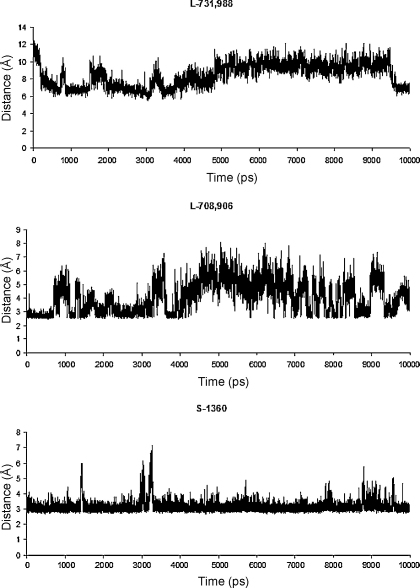
The distance between the carboxylate OE_2_ of Glu152 and NH_2_ of Arg199, versus simulation time for the IN proteins, uncomplexed or bound with DKA inhibitors.

The time evolution of the distances between Glu152 and Arg199 is given in Fig. 8. Compared with the relative position of Glu152 to Arg199 in the IN crystal structure, the Glu152 in IN bound with DKA inhibitors tends to engage an ion–pair interaction with Arg199 on α6 which is attached to the C-terminal. The distances between OE_2_ of Glu152 and NH_2_ of Arg199 in IN-S-1360, and IN-L-708,906 are 5.8 Å and 5.0 Å, respectively, implying formation of weak salt bridge interactions (Fig. 5); In IN bound L-731,988, Glu152 is also pushed away from the catalytic centre, but it is still too far away from R199 to form a salt bridge interaction. The distances between Glu152 and Arg199 in the minimized average structures over the last 500 ps of simulations are listed in Table 1. It can be seen that the order of the distance correlates with their potency, indicating that the formation of the additional salt bridge between Glu152 and Arg199 can greatly constrain the flexibility of the surface loop (residues 138–149) and therefore may account for their biological activities.

We therefore propose that the salt bridge between Lys159 and the acid group of the compounds is indispensable for the compounds to exhibit inhibition activities, but the order of potency of the DKA inhibitors depends on formation of widened loop conformation accompanied by formation of an additional ion–pair interaction between Glu152 and Arg199. Since the C-terminal domain of IN has been suggested to bind the viral DNA during the integration process [7], forming of the salt ion interaction between Glu152 and Arg199 may constrain the flexibility of an elbow kink on α6 which links the C-terminal to the catalytic core domain, thus preventing the target DNA substrate from accessing the IN.

Direct interactions between the DKA compounds and another two residues related to inhibitor resistance (Ser153 and Met154), were not reported in the average structures. We suggest that mutation of these two residues may induce a conformational change of the active site, in particular the reorientation of Glu152, such that the inhibition function of DKA inhibitors is interrupted.

### 3.7 Interpretation of the strand transfer activities

We calculated the averaged interaction energies over the last 500 ps of the MD simulation (Table 1). It can be seen that for Merck inhibitors, the interaction energies with IN are correlated well with the biological activities of DKA inhibitors. The interaction energy with L-731,988, which is −128 kcal/mol, is less than that of L-708,906, which is −139 kcal/mol. The order of the interaction energies of L-731,988 and L-708,906 are in agreement with experimental activities [53]. The interaction energy of S-1360 is greater than that of L-731,988; however, its energy is similar to that of L-708,906, in contrast to the experimental observation. The interaction energies are not correlated very well to the binding affinities, indicating the difference in the entropic contribution during the binding may not be neglected. It also needs to be noted that the interaction energies calculated here were the sum of van der Waals, Coulomb and H-bond terms, and that the cation–π interaction observed in S-1360 and IN is not well accounted by the standard force field. So the change of free energy obtained from experiment may be different from the interaction energies calculated here, and therefore the order of the binding affinities is not strictly correlated with the interaction energies. In saying that, the aim of our work was to correlate the potency order, rather than quantify the binding energy. The order of the interaction energy can be explained by the binding modes among different DKA inhibitors. The aromatic groups of the Merck inhibitors L-731,988, L-708,906 and also S-1360 can fit well into the hydrophobic cavities identified (Fig. 6). While nested in the hydrophobic cavity, the acidic part of these compounds forms salt bridge interactions with Lys159, therefore, exhibiting potent strand transfer activity. Merck inhibitor L-731,927 [19] (Fig. 2) has a phenyl group which could be accommodated in the hydrophobic pocket, due to the flexibility of ethyl group. Consequentially, it has comparable strand transfer activity compared with L-731,988 and L-708,906. However, for another Merck inhibitor with biphenyl substituent, L-731,942, the second benzene ring is too big to be accommodated in the hydrophobic cavity near Gln146 and as a result, its strand transfer inhibition concentration is of higher magnitude.

## 4 Conclusion

The structures of HIV-1 IN in complex with several representative DKA inhibitors, namely Merck inhibitors L-731,988, L-708,906 and Shionogi inhibitor S-1360, were studied by comparative molecular dynamics simulations. We found that the acidic end of all the DKA inhibitors studied formed favourable ionic interactions with Lys159. The keto–enol parts of these compounds were consistently coordinated to Mg, compatible with the putative metal sequestration model proposed by Grobler et al. and also the recently resolved crystal structure of prototype foamy virus (PFV) IN bound with two HIV-1 IN drugs. The enol oxygen of the DKA was hydrogen-bonded to two of the catalytic residues, Asp64 and Asp116, either directly or mediated by bridging water molecules identified throughout the MD simulations. This is in agreement with the experimental study where D64A and D116A mutants both confer resistance to the Merck inhibitors. The catalytic residue Glu152 formed a favourable ion–pair interaction with the negatively charged Arg199 on α6, when IN is complexed with either L-708,906 or S-1360. The complexation with Merck inhibitors and S-1360 significantly constrained the flexible surface loop into an extended or open conformation. The hydrophobic contact with several residues on the flexible surface loop (residues 138–149), including Ile141, Pro142, Asn155, as well as Gln146 and Gln148, with the aromatic ring of Merck inhibitors and S-1360 accounted for the stabilization of the flexible active site loop.

Our result has demonstrated that the Merck inhibitors and S-1360 share a similar IN binding mode, which involves chelating with the metal ion and forming a salt ion interaction with Lys159. The difference in binding affinities, however, may be interpreted by the formation of an additional salt bridge with R199, by reorientation of the catalytic residue Glu152 effectively constraining the flexibility of active site loop, residues 138–149. The interaction modes of DKA inhibitors, provided here, demonstrate that the dynamic aspects of complexation must not be ignored in structure-based inhibitor design. A complete understanding of the conformation adopted by the catalytic loop upon binding of DKA inhibitors would, indeed shed light on design of novel HIV-1 IN inhibitors.

## Figures and Tables

**Table 1:** Binding interactions between DKA and HIV-1 IN (averaged over last 500 ps).

IN complex	Interaction energy (kcal/mol)	Strand transfer (ST) IC_50_ (μM)	Hydrophobic contacts	Distance (Å)	
				E152-R199	K159-carboxylate or triazole
L-731,988	−128.4	0.17^a^	I141; Y143, N155, K156	8.9	4.5
L-708,906	−138.9	0.10^b^	V79, I141, P142, P145, Q146, S147; Q148, N155^d^, K156	5.0	2.6
S-1360	−137.7	0.02^c^	P142; P145, Q146, N155, K156	5.8	2.9

a Ref: [53].

b Ref: [17].

c Ref: [18].

d The nitrogen atom on the sidechain amide group of N155 also forms H-bond with L-708,906.

## References

[1] Dayam R., Neamati N. (2003). Small-molecule HIV-1 integrase inhibitors: the 2001–2002 update. Curr. Pharm. Des..

[2] Maurin C., Bailly F., Cotelle P. (2003). Structure–activity relationships of HIV-1 integrase inhibitors – enzyme–ligand interactions. Curr. Med. Chem..

[3] Parrill A.L. (2003). HIV-1 integrase Inhibition: binding sites structure–activity relationships and future perspectives. Curr. Med. Chem..

[4] Nair V. (2003). Novel inhibitors of HIV integrase: the discovery of potential anti-HIV therapeutic agents. Curr. Pharm. Des..

[5] Pommier Y., Johnson A.A., Marchand C. (2005). Integrase inhibitors to treat HIV/AIDS. Nat. Rev. Drug Discov..

[6] Engelman A., Mizuuchi K., Craigie R. (1991). HIV-1 DNA interaction – mechanism of viral-DNA cleavage and DNA strand transfer. Cell.

[7] Chen J.C.H., Krucinski J., Miercke L.J., Finer-More J.S., Tang A.H., Leavitt A.D., Stroud R.M. (2000). Crystal structure of the HIV-1 integrase catalytic core and C-terminal domains: a model for viral DNA binding. Proc. Natl. Acad. Sci. U. S. A..

[8] Engelman A., Bushman F.D., Craigie R. (1993). Identification of discrete functional domains of HIV-1 integrase and their organization within an active multimeric complex. EMBO (Eur. Mol. Biol. Organ) J..

[9] Vangent D.C., Vink C., Groeneger A.A.M.O., Plasterk R.H.A. (1993). Complementation between HIV integrase proteins mutated in different domains. EMBO (Eur. Mol. Biol. Organ) J..

[10] Buiacz G., Jaskólski M., Alexandratos J., Wlodrawer A., Merkek G., Katz R.A., Skalka A.M. (1995). High-resolution structure of the catalytic domain of avain sarcoma virus integrase. J. Mol. Biol..

[11] Rice P.A., Baker T.A. (2001). Comparative architecture of transposase and integrase complexes. Nat. Struct. Biol..

[12] Grobler J.A., Stillmock K., Hu B.H., Witmer M., Felock P., Espeseth A.S., Wolfe A., Egbertson M., Bourgeois M., Melamed J., Wai J.S., Young S., Vacca J., Hazuda D.J. (2002). Diketo acid inhibitor mechanism and HIV-1 integrase: implications for metal binding in the active site of phosphotransferase enzymes. Proc. Natl. Acad. Sci. U. S. A..

[13] Marchand C., Johnson A.A., Karki R.G., Pais G.C.G., Zhang X.C., Cowansage K., Patel T.A., Nicklaus M.C., Burke T.R., Pommier Y. (2003). Metal-dependent inhibition of HIV-1 integrase by beta-diketo acids and resistance of the soluble double-mutant (F185K/C280S). Mol. Pharmacol..

[14] Greenwald J., Le V., Butler S.L., Bushman F.D., Choe S. (1999). The mobility of an HIV-1 integrase active site loop is correlated with catalytic activity. Biochemistry.

[15] Jenkins T.M., Esposito D., Engelman A., Craigie R. (1997). Critical contacts between HIV-1 integrase and viral DNA identified by structure-based analysis and photo-crosslinking. EMBO (Eur. Mol. Biol. Organ) J..

[16] Esposito D., Craigie R. (1998). Sequence specificity of viral end DNA binding by HIV-1 integrase reveals critical regions for protein–DNA interaction. EMBO (Eur. Mol. Biol. Organ) J..

[17] Hazuda D.J., Felock P., Witmer M., Wolfe A., Stillmock K., Grobler J.A., Espeseth A., Gabryelski L., Schleif W., Blau C., Miller M.D. (2000). Inhibitors of strand transfer that prevent integration and inhibit HIV-1 replication in cells. Science.

[18] Billich A. (2003). S-1360 Shionogi-GlaxoSmithKline. Curr. Opin. Investig. Drugs.

[19] Hazuda D.J., Anthony N.J., Gomez R.P., Jolly S.M., Wai J.S., Zhuang L., Fisher T.E., Embrey M., Guare J.P., Egbertson M.S., Vacca J.P., Huff J.R., Felock P.J., Witmer M.V., Stillmock K.A., Danovich R., Grobler J., Miller M.D., Espeseth A.S., Jin L., Chen I.W., Lin J.H., Kassahun K., Ellis J.D., Wong B.K., Xu W., Pearson P.G., Schleif W.A., Cortese R., Emini E., Summa V., Holloway M.K., Young S.D. (2004). A naphthyridine carboxamide provides evidence for discordant resistance between mechanistically identical inhibitors of HIV-1 integrase. Proc. Natl. Acad. Sci. U. S. A..

[20] Fikkert V., Hombrouck A., Van Remoortel B., De Maeyer M., Pannecouque C., De Clercq E., Debyser Z., Witvrouw M. (2004). Multiple mutations in human immunodeficiency virus-1 integrase confer resistance to the clinical trial drug S-1360. AIDS.

[21] Zolopa A., Mullen M., Berger D., Ruane P., Hawkins T., Zhong L., Chuck S., Enejosa J., Kearney B., Cheng A. (2007). The HIV integrase inhibitor GS-9137 demonstrates potent ARV activity in treatment-experienced patients. 14th Conf. RetroVir. Opportun. Infect.

[22] Evering T.H., Markowitz M. (2008). Raltegravir: an integrase inhibitor for HIV-1. Expert Opin. Invest. Drugs.

[23] Marinello J., Marchand C., Mott B.T., Brain A., Thomas C.J., Pommier Y. (2008). Comparison of Raltegravir and Elvitegravir on HIV-1 integrase catalytic reactions and on a series of drug-resistant integrase mutants. Biochemistry.

[24] Buzón M.J., Dalmau J., Puertas M.C., Puig J., Clotet B., Martinez-Picado J. (2010). The HIV-1 integrase genotype strongly predicts Raltegravir susceptibility but not viral fitness of primary virus isolates. AIDS.

[25] Grobler J.A., Stillmock K., Miller M.D., Hazuda D.J. (2008). Mechanism by which the HIV integrase active-site mutation N155H confers resistance to Raltegravir. Antivir. Ther..

[26] Goldgur Y., Craigie R., Cohen G.H., Fujiwara T., Yoshinaga T., Fujishita T., Suginoto H., Endo T., Murai H., Davies D.R. (1999). Structure of the HIV-1 integrase catalytic domain complexed with an inhibitor: a platform for antiviral drug design. Proc. Natl. Acad. Sci. U. S. A..

[27] Hare S., Gupta S.S., Valkov E., Engelman A., Cherepanov P. (2010). Retroviral intasome assembly and inhibition of DNA strand transfer. Nature.

[28] Fitzkee N.C., Masse J.E., Shen Y., Davies D.R., Bax A. (2010). Solution conformation and dynamics of the HIV-1 integrase core domain. J. Biol. Chem..

[29] Sotriffer C.A., Ni H., McCammon J.A. (2000). HIV-1 integrase inhibitor interactions at the active site: prediction of binding modes unaffected by crystal packing. J. Am. Chem. Soc..

[30] Keseru G.M., Kolossvary I. (2001). Fully flexible low-mode docking: application to induced fit in HIV integrase. J. Am. Chem. Soc..

[31] Lins R.D., Adesokan A., Soares T.A., Briggs J.M. (2000). Investigations on human immunodeficiency virus type 1 integrase/DNA binding interactions via molecular dynamics and electrostatics calculations. Pharmacol. Ther..

[32] Brigo A., Lee K.W., Mustata G.I., Briggs J.M. (2005). Comparison of multiple molecular dynamics trajectories calculated for the drug-resistant HIV-1 integrase T66I/M154I catalytic domain. Biophys. J..

[33] Lee M.C., Deng J., Briggs J.M., Duan Y. (2005). Large-scale conformational dynamics of the HIV-1 integrase core domain and its catalytic loop mutants. Biophys. J..

[34] Schames J.R., Henchman R.H., Siegel J.S., Sotriffer C.A., Ni H.H., McCammon J.A. (2004). Discovery of a novel binding trench in HIV integrase. J. Med. Chem..

[35] Ni H.H., Sotriffer C.A., McCammon J.A. (2001). Ordered water and ligand mobility in the HIV-1 integrase-5CITEP complex: a molecular dynamics study. J. Med. Chem..

[36] Barreca M.L., Lee K.W., Chimirri A., Briggs J.M. (2003). Molecular dynamics studies of the wild-type and double mutant HIV-1 integrase complexed with the 5CITEP inhibitor: mechanism for inhibition and drug resistance. Biophys. J..

[37] Brigo A., Lee K.W., Fogolari E., Mustata G.I., Briggs J.M. (2005). Comparative molecular dynamics simulations of HIV-1 integrase and the T66I/M154I mutant: binding modes and drug resistance to a diketo acid inhibitor. Proteins: Struct. Funct. Bioinform..

[38] Nunthaboot N., Pianwanit S., Parasuk V., Ebalunode J.O., Briggs J.M., Kokpol S. (2007). Hybrid quantum mechanical/molecular mechanical molecular dynamics simulations of HIV-1 integrase/inhibitor complexes. Biophys. J..

[39] Aleves C.N., Marti S., Castillo R., Andres J., Moliner V., Inaki Tunon E., Silla A. (2008). Quantum mechanic/molecular mechanic study of the wild-type and N155S mutant hiv-1 integrase complexed with diketo acid. Biophys. J..

[40] Aleves C.N., Marti S., Castilio R., Andres J., Moliner V., Inaki Tunon, Silla E.A. (2007). Quantum mechanics/molecular mechanic study of the protein–ligand interaction for inhibitors of HIV-1 integrase. Chem. Eur. J..

[41] Aleves C.N., Marti S., Castilio R., Andres J., Moliner V., Inaki Tunon, Silla E. (2007). Calculation of binding energy using BLYP/MM for HIV-1 integrase complexed with the S-1360 and two analogues. Bioorg. Med. Chem..

[42] Frisch M.J., Trucks G.W., Schlegel H.B., Scuseria G.E., Robb M.A., Cheeseman J.R., Zakrzewski V.G., Montgomery J.A., Stratmann R.E., Burant J.C., Dapprich S., Millam J.M., Daniels A.D., Kudin K.N., Strain M.C., Farkas O., Tomasi J., Barone V., Cossi M., Cammi R., Mennucci B., Pomelli C., Adamo Clifford C.S., Ochterski J., Petersson G.A., Ayala P.Y., Cui Q., Morokuma K., Salvador P., Dannenberg J.J., Malick D.K., Rabuck A.D., Raghavachari K., Foresman J.B., Cioslowski J., Ortiz J.V., Baboul A.G., Stefanov B.B., Liu G., Liashenko A., Piskorz P., Komaromi I., Gomperts R., Martin R.L., Fox D.J., Keith T., Al-Laham M.A., Peng C.Y., Nanayakkara A., Challacombe M., Gill P.M.W., Johnson B., Chen W., Wong M.W., Andres J.L., Gonzalez C., Head-Gordon M., Replogle E.S., Pople J.A. (2001). Gaussian 98.

[43] Goldgur Y., Dyda F., Hickman A.B., Jenkins T.M., Craigie R., Davis D.R. (1998). Three new structures of the core domain of HIV-1 integrase: an active site that binds magnesium. Proc. Natl. Acad. Sci. U. S. A..

[44] Maignan S., Guilloteau J.P., Zhou-Liu Q., Clement-Mella C., Mikol V. (1998). Crystal structures of the catalytic domain of HIV-1 integrase free and complexed with its metal cofactor: high level of similarity of the active site with other viral integrases. J. Mol. Biol..

[45] Brooks C.L., Bruccoleri R.E., Olafson B.D., States D.J., Swaminathan S., Karplus M. (1983). CHARMM: a program for macromolecular energy minimization, and dynamics calculations. J. Comput. Chem..

[46] MacKerell A.D., Bashford D., Bellott M., Dunbrack R.L., Evanseck J.D., Field M.J., Fischer S., Gao J., Guo H., Ha S., Joseph-McCarthy D., Kuchnir L., Kuczera K., Lau F.T.K., Mattos C., Michnick S., Ngo T., Nguyen D.T., Prodhom B., Reiher W.E., Roux B., Schlenkrich M., Smith J.C., Stote R., Straub J., Watanabe M., Wiorkiewicz-Kuczera J., Yin D., Karplus M. (1998). All-atom empirical potential for molecular modelling and dynamics studies of proteins. J. Phys. Chem. B.

[47] Spassov V.Z., Yan L. (2008). A fast and accurate computational approach to protein ionization. Protein Sci..

[48] Jorgensen W.L., Chandrasekhar J., Madura J.D., Impey R.W., Klein M.L. (1983). Comparison of simple potential functions for simulating liquid water. J. Chem. Phys..

[49] Brooks C.L., Karplus M. (1989). Solvent effects on protein motion and protein effects on solvent motion – dynamics of the active-site region of lysozyme. J. Mol. Biol..

[50] Huang M., Richards W.G., Grant G.H. (2005). Diketo acid HIV-1 integrase Inhibitors: an ab initio study. J. Phys. Chem. A.

[51] Woodruff M., Plya J.B. (1975). Pyrazolidine-3, 5-diones with heterocyclic substituents. IV. Ionization constants. Aust. J. Chem..

[52] Wielens J., Headey S.J., Jeevarajah D., Rhodes D.I., Deadman J., Chalmers D.K., Scanlon M.J., Parker M.W. (2010). Crystal structure of the HIV-1 integrase core domain in complex with sucrose reveals details of an allosteric inhibitory binding site. FEBS Lett..

[53] Wai J.S., Egbertson M.S., Payne L.S., Fisher T.E., Embrey M.W., Tran L.O., Melamed J.Y., Langford H.M., Guare J.P., Zhuang L., Grey V.E., Vacca J.P., Holloway M.K., Naylor-Olsen A.M., Hazuda D.J., Felock P.J., Wolfe A.L., Stillmock K.A., Schleif W.A., Gabryelski L.J., Young D.S. (2000). 4-Aryl-2,4-dioxobutanoic acid inhibitors of HIV-1 integrase and viral replication in cells. J. Med. Chem..

