# Supplementary material for: Binding modes of diketo-acid inhibitors of HIV-1 integrase: A comparative molecular dynamics simulation study
Source: J Mol Graph Model. 2011 Jun;29(7):956–64. doi: 10.1016/j.jmgm.2011.04.002 (PMC3101338; doi:10.1016/j.jmgm.2011.04.002)
Supplement: Supplementary file 1 [file mmc1.doc]

**Table S1** Mutations in IN that confer resistance to DKA and their derivatives.

| DKA and derivatives | Mutations |
| --- | --- |
| L-731,988 | T66I/M154I, T66I /S153Y , N155S |
| L-708,906 | T66I/M154I, T66I /S153Y , N155S |
| L-870,810 | N155S |
| S-1360 | T66I, Q146K, S153A |
| Raltegravir (MK-0518) | G140A/Q148K, T66I, S153Y, Y143R, N155H |
| Elvitegravir (GS-9137) | Q148K, T66I, S153Y |


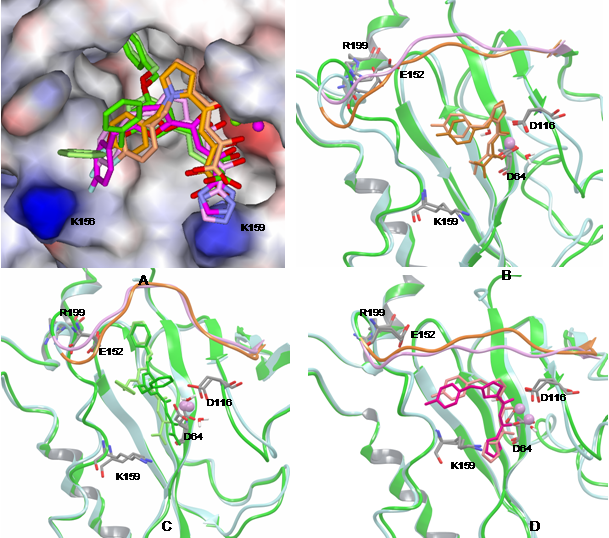


**Figure S1**. IN bound with DKA inhibitors from conformations averaged over 8.5-9ns (in cyan) and 9.5-10ns (in green). The two structural water molecules which are involved in Mg ion coordination are shown in tube mode. (A) The surface representation of the DKA binding site (B) IN bound with L-731,988 (C) IN bound with L708,906 (D) IN bound with S-1360.


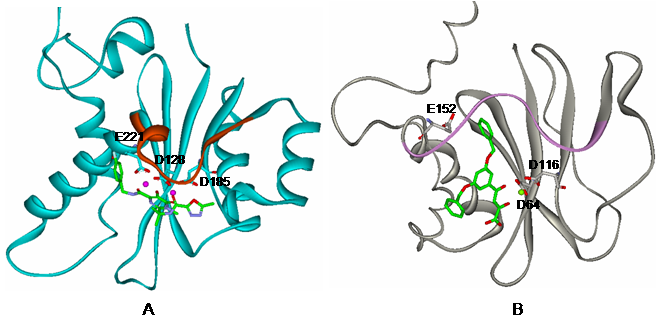


**Figure S2**. The catalytic loop in HIV-1 IN is opened with the DKA binding. (A) Prototype Foamy Virus (PFV) Integrase in complex with an HIV-1 drug, Raltegravir (pdb code: 3OYA). Raltegravir is shown in tube mode in green, the catalytic loop is shown in red; the catalytic triad is formed by D128, D185 and E221. (B) HIV-1 IN in complex with a Merck inhibitor L-708,906 from MD simulation. L-708,906 is shown in green, and the catalytic loop is shown pink; the catalytic triad is formed by D64, D116 and E152.


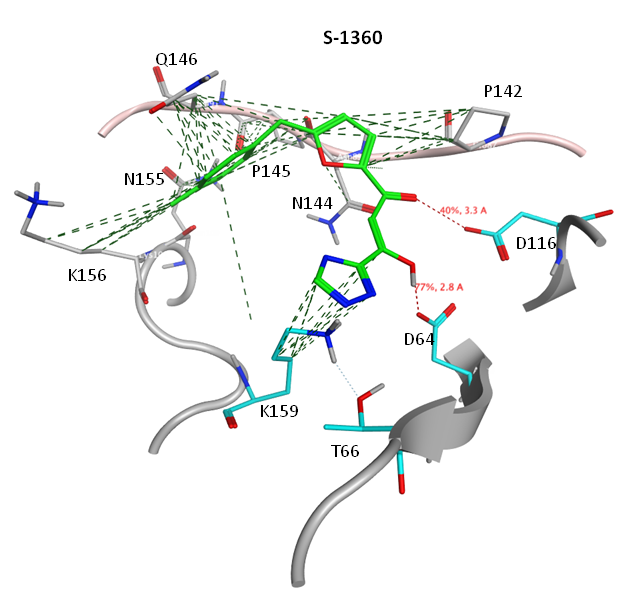


**A**


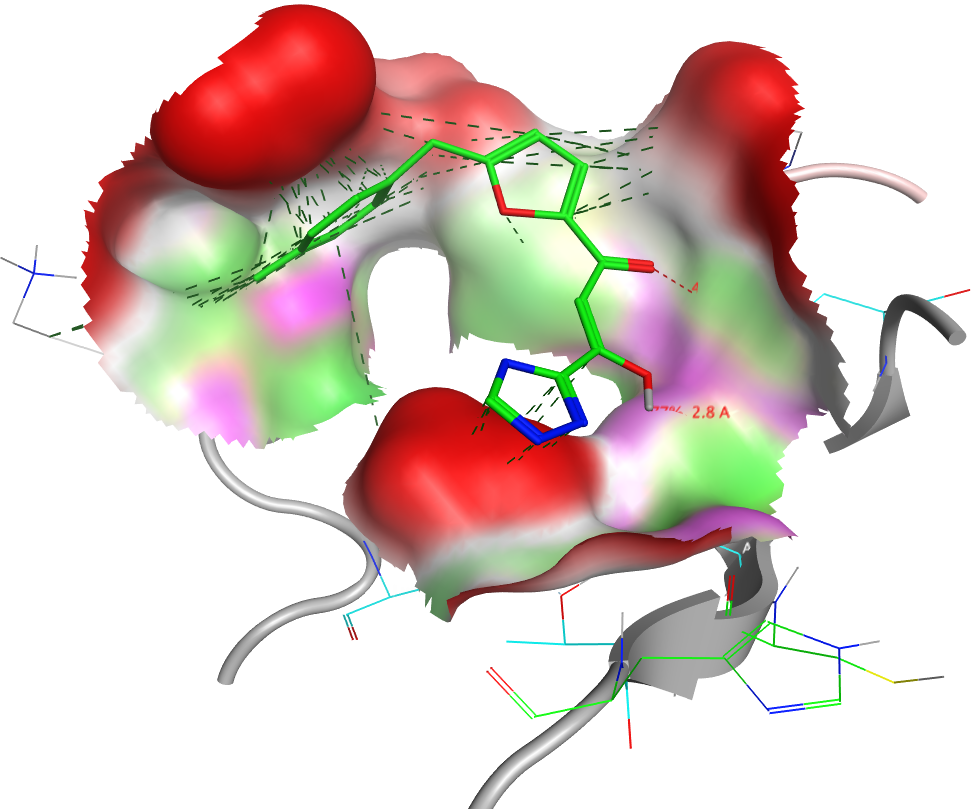


**B**

**Figure S3.** (A) The hydrophobic interactions (shown in green line) between S-1360 and IN. S-1360 is shown in stick mode. The carbon atoms in S-1360 are shown in green. (B) Binding cavity of S-1360. The colour scheme in the surface representation is: the polar regions are shown in pink, the hydrophobic regions are shown in green and the exposed regions are shown in red.


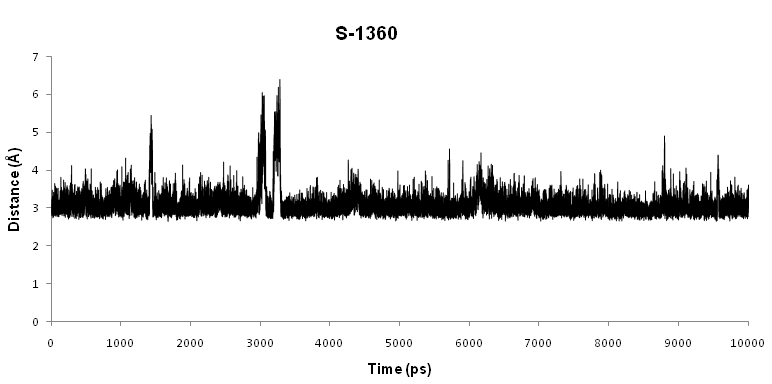


**A**


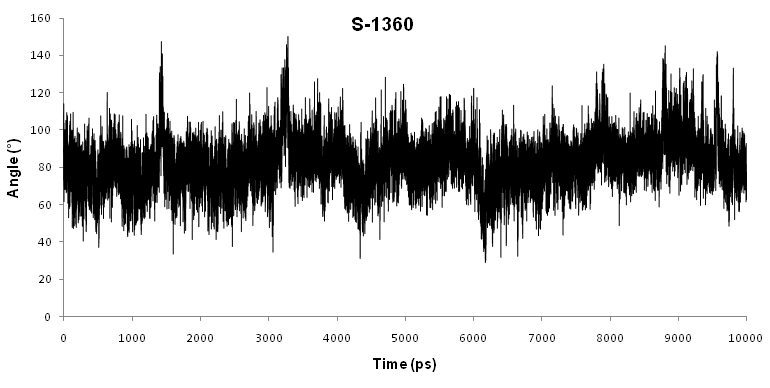


**B**

**Figure S4 (A).** The distance between the ammonia NZ of Lys159 and the centroid of the triazole ring of S-1360, versus simulation time for the IN in complex with S-1360. (B). The angle formed by the ammonia NZ of Lys159, the centroid and N1 atom of the triazole ring of S-1360, versus simulation time for the IN in complex with S-1360.
